# Complete genome sequence of *Neobacillus* strain fa239, isolated from soil in Mie, Japan

**DOI:** 10.1128/mra.00837-25

**Published:** 2025-09-24

**Authors:** Ayumi Tanimura

**Affiliations:** 1 Institutes of Innovation for Future Society, Nagoya University 12965, Nagoya, Japan; Portland State University, Portland, Oregon, USA

**Keywords:** Bacillaceae, complete genome sequence

## Abstract

This report presents the complete genome sequence of *Neobacillus* strain fa239, isolated from soil in Mie, Japan. The genome consists of two circular contigs totaling 6,133,609 bp, with a GC content of 39.3%. Sequencing was performed on the PacBio Revio platform.

## ANNOUNCEMENT

Strain fa239 was isolated from a surface soil sample (0–5 cm depth) collected at Mitakien Higashi Park in Komono Town, Mie Prefecture, Japan (35.018539°N, 136.514897°E), a non-allophanic Andosol site. The sample was suspended in sterile saline, serially diluted, and plated on an in-house agar medium containing inorganic salts, trace elements, and a C1 carbon source. Plates were incubated aerobically at 25°C for 3 days. A single colony was isolated and purified by repeated streaking on YM agar (1).

Taxonomic identification was performed by extracting the 16S rRNA gene sequence from the complete genome assembly and comparing it to type strain sequences using BLASTn against the NCBI nucleotide database (2). The sequence showed 98.45% identity to that of *Neobacillus driksii* (GenBank accession number PP849388.1).

For genome sequencing, strain fa239 was cultured in YM broth at 25°C overnight. Genomic DNA was extracted using the Genomic-tip 20G Kit (Qiagen). No intentional DNA shearing was performed. Short fragments were removed using the Short Read Eliminator XS Kit (PacBio). Library preparation was performed with the SMRTbell Prep Kit 3.0 and the SMRTbell gDNA Sample Amplification Kit (PacBio) according to the manufacturer’s protocols. Sequencing was carried out on a PacBio Revio platform using the Revio Polymerase Kit. A total of 46,623 reads were obtained (mean 5.3 kb, N50 5.4 kb; 247.8 Mb). Quality filtering with Filtlong v0.2.1 (3) removed reads shorter than 1,000 bp and the lowest quality, 10%, yielding ≥95.5% of bases with Q ≥ 30. Genome coverage was estimated at 40.4×.

HiFi reads were generated using SMRT Link v11.0 (4). Adapter trimming was performed with lima v2.12.0 (PacBio) (5), and duplicate reads were removed using pbmarkdup v1.0.3 (PacBio) (6). No additional error correction was performed, as PacBio HiFi reads are already highly accurate. Genome assembly was performed using Flye v2.9.3-b1797 (7), yielding two circular contigs totaling 6,133,609  bp (N50 5,706,330 bp; GC 39.3%). These were a chromosome (5,706,330 bp) and a plasmid (427,279 bp). Circularization of both contigs was verified manually by trimming overlapping ends to produce a seamless circular genome. For the deposited record, assembler-derived coordinates were retained; thus, the chromosomal dnaA gene is at 5,497,224–5,498,570 rather than coordinate 1. The smaller circular contig was deposited unrotated because no unambiguous plasmid replication initiation gene (e.g., rep) was identified. Assembly graphs were visualized with Bandage v0.8.1 (8), and genome completeness and contamination were assessed with CheckM2 v1.2.2 (9), which indicated 100.0% completeness and 9.16% contamination, likely reflecting closely related strains or reference database limitations.

Gene prediction used Prokka v1.14.6 (10), followed by functional annotation with DIAMOND v2.1.6 (11) against the KEGG and GO databases (12, 13). Annotation was also performed using DFAST v1.2.18 (14). The complete genome comprises 5,869 predicted coding sequences, 54 rRNA genes, and 160 tRNA genes. A single plasmid replicon was identified. This complete genome sequence will support taxonomic and phylogenetic studies of *Neobacillus*-related environmental bacteria. Default parameters were used unless otherwise noted.

## Data Availability

The Whole Genome Shotgun project has been deposited in the DDBJ/ENA/GenBank under accession nos. AP041611.1 (chromosome) and AP041612.1 (plasmid). Raw sequence reads are available in the DDBJ Sequence Read Archive (DRA) under accession no. DRR708776, associated with BioProject accession no. PRJDB35415 and BioSample accession no. SAMD01574884.
